# Role of Food Antioxidants in Modulating Gut Microbial Communities: Novel Understandings in Intestinal Oxidative Stress Damage and Their Impact on Host Health

**DOI:** 10.3390/antiox10101563

**Published:** 2021-09-30

**Authors:** Muhammad Shahid Riaz Rajoka, Rohit Thirumdas, Hafiza Mahreen Mehwish, Muhammad Umair, Mohsin Khurshid, Hafiz Fakhar Hayat, Yuthana Phimolsiripol, Noelia Pallarés, Francisco J. Martí-Quijal, Francisco J. Barba

**Affiliations:** 1 Health Science Center, School of Pharmaceutical Science, Shenzhen University, Shenzhen 518060, China; shahidrajoka@yahoo.com (M.S.R.R.); mahreen.mehwish@yahoo.com (H.M.M.); 2 Food and Feed Immunology Group, Laboratory of Animal Food Function, Graduate School of Agricultural Science, Tohoku University, Sendai 980-8572, Japan; 3 College of Chemistry and Environmental Engineering, Shenzhen University, Shenzhen 518060, China; umair_uaf@hotmail.com; 4 College of Food Science and Technology, PJTSAU, Hyderabad 500030, India; rohit.thirumdas@gmail.com; 5 Department of Microbiology, Government College University, Faisalabad Government College University, Faisalabad 38000, Pakistan; mohsinkhurshid@gcuf.edu.pk (M.K.); fakharuaf2013@gmail.com (H.F.H.); 6 Faculty of Agro-Industry, Chiang Mai University, Chiang Mai 50100, Thailand; yuthana.p@cmu.ac.th; 7 Department of Preventive Medicine and Public Health, Food Science, Toxicology and Forensic Medicine, Faculty of Pharmacy, Universitat de València, Avda. Vicent Andrés Estellés, 46100 València, Spain; Noelia.pallares@uv.es (N.P.); francisco.j.marti@uv.es (F.J.M.-Q.)

**Keywords:** antioxidants, food additive, gut microbiota, vitamins, polyphenol, bioactive peptides

## Abstract

Dietary components have an important role on the structure and function of host gut microbial communities. Even though, various dietary components, such as carbohydrates, fats, proteins, fibers, and vitamins, have been studied in depth for their effect on gut microbiomes, little attention has been paid regarding the impact of several food antioxidants on the gut microbiome. The long-term exposure to reactive oxygen species (ROS) can cause microbial dysbiosis which leads to numerous intestinal diseases such as microbiota dysbiosis, intestinal injury, colorectal cancers, enteric infections, and inflammatory bowel diseases. Recently, it has been shown that the food derived antioxidant compounds might protect the host from intestinal oxidative stress via modulating the composition of beneficial microbial species in the gut. The present review summarizes the impact of food antioxidants including antioxidant vitamins, dietary polyphenols, carotenoids, and bioactive peptides on the structure as well as function of host gut microbial communities. Several in vitro, animal model, and clinical studies indicates that food antioxidants might modify the host gut microbial communities and their health status. However, still further clarification is needed as to whether changes in certain microbial species caused by food additives may lead to changes in metabolism and immune function.

## 1. Introduction

The gut microbiota is considered as an extremely energetic metabolic organ owing to the complex activity of their microbial communities, having the ability to offer protective, immunologic and metabolic functions as outcome of active mutualistic associations amongst the microbial and host species [1]. Hence, any changes in the composition of gut microbiota ultimately affect the host health [2]. The gut microbiota can be considered as a complex ecosystem comprising of almost 100 trillion of microorganisms of more than 1000 species. For quite a few years, the gut microbiota was considered as a forgotten organ but now it is considered as an active metabolizing organ instead of just secretion organ [3]. The gut colonization occurred during first two years of life. It is assuming that the infant gut is sterile, when fetus is in the uterus. Immediately, after the birth of infants it starts receiving the microbes. The initial colonization of microbe and its intestinal distribution are thought to be beneficial by coordinating the host’s immune system [4].

Therefore, any alteration in the microbiota composition is linked with numerous metabolic disorders [5,6]. The most abundant phyla of gut microbiota are Firmicutes and Bacteroidetes which have been usually separated from the host via epithelial layer [7]. Furthermore, the colonization and spatial organization of gut microbiota are not uniform along the gastrointestinal tract. The highest microbial population is found in colon estimating 10^13^ bacteria/gram of colon content, along with a broad range of enzymes, which are helpful for dietary components biotransformation [8,9]. After digestion, the microbial biotransformation of dietary components occurred in lumen where microbial fermentation enhances the production of specific metabolites with health beneficial effect [10]. Hence, the microbiota composition and metabolites production vary among the individual and within them throughout the whole life. In short, the gut microbiota establishes communally beneficial connection which in turn helping the host by stimulating various physiological/biochemical functions via completing the complex metabolic process along with the host immune system regulation [11].

It is very well understood that the gut microbiota plays an important role in the metabolism of food derived compounds that are undigested in the upper part of the digestive tract. On the other hand, more research has to be conducted to understand the gut microbiota impact on host health, especially their role in food metabolism and xenobiotics transformation [12]. Furthermore, the gut microbiota contains numerous enzymes (α-rhamnosidase, β-glucuronidase, β-glucosidase, sulfatase and various esterases) having the ability to hydrolyze glycosides, glucuronides, sulfates, amides, esters and lactones [13,14]. Gut microbiota breakdown the complexes involving proteins/carbohydrate/polyphenols into vitamins and amino acids, which are involved in a wide variety of host physiological function such as host energy metabolism and immunity [15].

Untreated vegetables contain substances having antioxidant properties with the ability to reduce the undesirable effect of redox process, which every cell experiences due to its biochemical reaction. Therefore, the vegetable-based diet such as Mediterranean diet, vegetarian, and vegan diet carry large quantities of these substances as compared with other diets such as Western diet and cafeteria diet often taken by people [16,17,18]. Furthermore, the processed food contains numerous food additive having negative effect on intestine, gut microbiota, liver, and nervous system of host due to oxidative stress [19]. The antioxidant substances can inhibit the oxidative stress mediated by free radicals as well as their toxic effect on the host body.

Here in this review, several factors that might disturb the microbiota composition are discussed, especially focused on the dietary elements which exert significant impact on microbiota composition and function. Although numerous investigations have shown the impact of main dietary derived elements on the gut microbiota composition, the consequence of minor diet derived components including antioxidant compounds on composition of gut microbiota have been fewer explored.

## 2. Composition of Gut Microbiota and Their Impact on Host Health 

**Figure 1:**
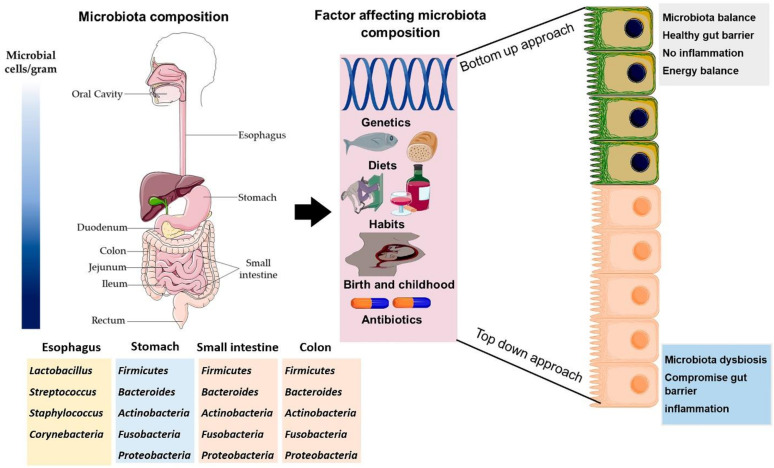
The microbiota composition throughout the length of gastrointestinal tract. Factor affecting the composition of gut microbiota throughout the life and consequence on host health. The sequence of microbiota distribution in gastrointestinal tract is oral cavity < stomach < duodenum < jejunum < colon. The image was created by using the service provide by Servier Medical Art (Suresnes, France) under license Creative Common Attribution 3.0 France.

The gut microbiota composition greatly varies among the location, length, and depth of host gastrointestinal tract, hence establishing regional precise microbial communities. Briefly, the quantity of microbial cells increased along with gut length [20] (Figure 1).

The Firmicutes and Bacteroidetes are two dominant phyla accounting for almost 90% population of total gut microbiota [21]. The proportion of these two dominant phyla can vary due to numerous intrinsic/extrinsic factors, but it generally remains similar in most of the healthy organisms [22]. Other phyla contributing to composition of host gut microbiota are Actinobacteria, Fusobacteria, Proteobacteria, Verrucomicrobia, and few species from Archaea domain. Furthermore, the *Escherichia*, *Bifidobacterium*, *Bacteroides*, *Clostridium*, *Streptococcus*, *Ruminococcus*, and *Lactobacillus* are the most important recognized genera of host gut microbiota [23].

The involvement of microbiota to host physiology is usually associated with microbial metabolism [24]. The microbial species in gut microbiota decompose the complex nutrients such as carbohydrate, protein, and fat, which reaching to the lower part of gut. For instance, numerous microbial associated enzymes are involved in the metabolism of these nutrients which ultimately play an important role in numerous host metabolic functions [25]. The gut microbiota plays a vital role in cyclic fission, hydrolysis, deamination, reduction, and dehydrogenation of complex nutrient to generate variety of metabolites (short chain fatty acids, hydrogen, alcohols, carbon dioxide, ammonia, choline derivatives, branched-chain fatty acids, phenols and indoles, and amines) which might be absorbed to blood and act throughout the host body [26,27,28]. Amongst these metabolites, the short chain fatty acids showed excellent ability to impact the preservation of colonic epithelial cells as these cells use them as an energy source [29]. Furthermore, the amino acid and lipid catabolism exert significant impact on composition of gut microbiota which in turn influence the host health [29].

In addition, gut microbiota plays a very important role in the development of immune system via influencing the physiology and gene expression of several cells in the host body [30]. The microbiota and immune cells interaction lead to creation of various signals that will encourage the differentiation of immune cell, maturation of immune organ and the development of other immune functions. Microbiota also can regulate some immune functions of host body including enhancement of macrophages phagocytic activity, generation of antibodies, and T cells differentiations [31]. Furthermore, the gut microbiota plays an important role in maintaining the integrity of epithelial cells as well as cell to cell connection which ultimately leads to restoration of epithelial damaged cell and enterocytes [32]. Hence, the gut microbiome has the ability to produce a wide range of functional genes and metabolite, being involved in maintenance of innate and adaptive immune functions which in turn directly contribute to the hosts health.

## 3. Impact of Diet on Composition of Gut Microbiota

The diet habits exert a significant impact on the microbiota composition. For instance, the consumption of plant-based protein enhances the composition of *Enterococcus*, *Bifidobacterium*, and *Lactobacillus* while it decreased the composition of *Escherichia coli*, *Bacteroides*, and *Clostridium* perfringens [33,34]. In addition, the consumption of plant-based protein enhanced the production of short chain fatty acids which in turn exerts anti-inflammatory effect during obesity and help in maintaining the mucosal barrier [35]. On the other hand, the consumption of animal-based protein increased the population of *Ruminococcus*, *Bacteroides*, *Bilophila*, and *Alistipes* while reducing the abundance of *Bifidobacterium* [36].

Zhu et al. [37] described that the consumption of inulin in the diet can modulate the composition of gut microbiota. They found that short-chain inulin reduces the proportion of *Lachnospiraceae*, *Bacteroidales* S24-7, *Ruminococcaceae* and *Lactobacillus* in intestinal tract microbiota, which leads to weight loss. One of the main products in Mediterranean diet is olive oil. Olive oil can modulate microbiota, having beneficial effects for the consumer [37]. In this sense, Gavahian et al. described the positive effects of olive oil on gut microbiota, preventing some non-communicable diseases such as diabetes or cardiovascular diseases. This effect is mainly due to the high presence of polyphenols in this matrix, but also to other compounds such as carotenoids or oleic acid [38]. Moreover, Žugčić et al., reviewed different beneficial effects of olive oil extracts, such as prevention of chronical diseases, mainly due to their antioxidant activity and then the reduction of oxidative stress [39].

The consumption of fiber rich diet enhances the abundance of *Lachnospiraceae*, *Bifidobacterium*, and *Prevotellaceae* while reducing the population of *Lactobacilli* and *Porphyromonadaceae*. Along with changing the microbiota composition, the consumption of fiber rich diet improves the production of short chain fatty acids, secretion of insulin, and glucose tolerance, while it reduces the weight gain and production of endogenous glucose [40]. Almost the 40 g of total intake carbohydrate (oligosaccharide, polysaccharide, and starch) in the diet usually reaches the colon daily by avoiding gastrointestinal digestion [41]. Recent studies revealed that changing the type as well as amount of carbohydrate nearly about four weeks showed strong impact on the composition of host microbiota [42].

**Figure 2:**
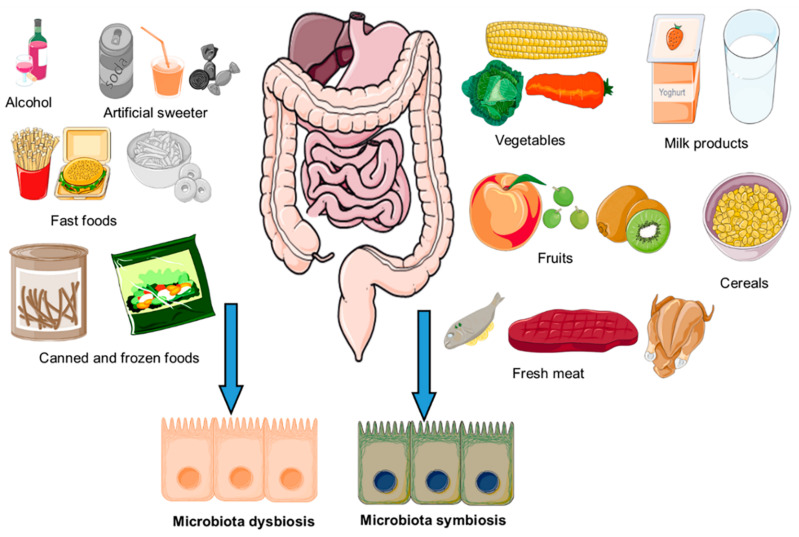
Dietary components that may affect the composition of microbiota leading to the gut symbiosis or dysbiosis. The image was created by using the service provide by Servier Medical Art under license Creative Common Attribution 3.0 France.

The fatty acids play a very important role in modulating the structural configuration of microbiota. For instance, the consumption of high fat diet reduced the abundance of *Eubacterium rectale*, *Clostridium coccoides*, *Bifidobacterium* and *Bacteroides* [43,44]. Moreover, it has been seen the beneficial effects of high-added-value compounds on gut microbiota, especially for omega-3 polyunsaturated fatty acids (PUFA), which improves the intestinal homeostasis and influences metabolic disfunction [45,46]. Furthermore, the consumption of PUFA play an important role in adherence of gut microbiota to mucosal surface and gut bacteria growth [47]. The impact of dietary factor on composition of host gut microbiota has been schematically illustrated in Figure 2. It was shown that the amount and type of diet drastically change the composition of gut microbiota. Therefore, the focus of present review is on the antioxidant dietary compounds and their impact on the composition as well as function of gut microbiota.

## 4. Oxidative Stress 

The free radicals are unstable and unwanted byproduct of cellular metabolism having unpaired electrons. This term needs to be clarified due to the connection between reactive oxygen species and free radicals is not entirely comprehensive and similar, but on the other hand ROS are more communal in the tendency of numerous disorders [48]. The ROS are generated through the continuous cell respiration process; therefore, the mitochondria might be considered as a primary source for ROS production in most of the organisms [49]. The ROS can be divided into free ROS (alkoxyl radical, peroxyl radical, hydroxyl radical, and superoxide) and non-free ROS (organic hydroperoxides, oxygen, chloramines, hydrogen peroxide, and hypochlorous acid) depending on the electron’s status. Amongst them, the hydroxyl radical is considered as the most reactive ROS species, while on the other hand hydrogen peroxide and hydroxyl radical are considered as important redox signal substances such as mitochondrial electron transport chain and NADPH oxidase [50,51]. The generation of ROS is a complex process. For example, endogenic ROS might attain self-balance state, while on the other hand the exogenic ROS generation required society to be aware of the hazard factors (Table 1).

## 5. Intestinal Oxidative Stress 

The oxidative injury happens when the antioxidant system does not eliminate the extra ROS from the host body. The oxidative stress mainly damages to macromolecules such as nucleic acid, proteins and lipids [58]. Proteins are the most common target for ROS attack as they are the most basic components of tissue and organs, being responsible for important physiological functions of host [59]. The ROS attack modify the amino acid residue, cross link the proteins and destroy the structure and peptide chain of targeted protein molecule [60]. The lipid peroxidation of biomembrane deserve more attention in oxidative stress damage because of solid attraction amongst reactive oxygen species and unsaturated fat. Furthermore, the lipid peroxidation can promote the destruction of normal cell function by changing its structure, biofilm fluidity, and cell wall permeability [61]. The oxidative stress is directly related to DNA damage and its bases modification. For example, the superoxide anion, hydroxyl radical, and hydrogen peroxide have been commonly involved in the DNA oxidative damage. The ROS are one of the most important factors for mutagenic, carcinogenic, and teratogenic effect [62,63]. Briefly, the potential foreign substances in intestine causing oxidative stress might target macromolecules (DNA, protein, and lipid) at molecular level which ultimately leading to macroscopic intestinal disease.

## 6. Oxidative Stress Modification through Nutrients and Microbiota

The symbiosis association host-microbe in intestine determine the oxidative stress level in host intestine, which might be influenced by the balance of gut microbiota [64]. For example, the *Lactobacillus brevis* 23,017 shelter the mucosal barrier by modifying the tight junction protein as well as prevent the oxidative stress along with inflammation via NF-κB and MAPK signaling pathways in mercury induced experimental mouse model [65]. The *Bacillus* SCo6 protect the host intestinal barrier via alleviating the production of intestinal oxidative stress in experimental IEC-6 cells model and rat model [66]. The supplementation of *Akkermansia muciniphila* can be a helpful strategy to reduce the production of oxidative stress along with the modulation of gut microbiota in streptozotocin-induced diabetic experimental rats’ model [67]. Furthermore, the consumption of *Lactobacillus plantarum* together with *Macleaya cordata* extract reduced the production of intestinal oxidative stress and protect the intestinal mucosal barrier in goat [68]. Hence, it is concluded that the consumption of probiotic bacteria might act as biological barrier to produce ROS, inhibiting the ROS producing enzymes, stimulating the antioxidant enzymes, enhancing the production of antioxidant metabolites and regulating antioxidant signaling pathways and gut microbiota [69,70].

Moreover, the indirect regulation of gut microbiota composition might be considered as another biological possible mechanism of antioxidant natural compounds and nutrients. The antioxidant natural compounds/nutrients alter the abundance and composition of gut microbiota, which ultimately reduces the production of ROS, activating the antioxidant enzymes and signaling pathways [71,72]. For example, the consumption of polypeptides reduced Firmicutes richness along with increasing the abundance of Bacteroidetes in hypoxic-ischemic-induced oxidative stressed rats. Moreover, these polypeptides improved the susceptibility to bacterial infections, increased the synthesis of antibiotics, and regulated inflammation, barrier dysfunction and oxidative stress [73]. It has been verified that *Salmonella typhimurium* can induce gastroenteritis, ileal injury, diarrhea, and intestinal flora disorder. However, the consumption of polyphenol reverses the ileal injury via regulating the intestinal flora, antioxidant enzyme activity, and proteins of tight junction in mice model [56]. The glucosinolates from Brassicaceae family are converted into isothiocyanates via myrosinases, a gut microbial enzyme, which then stimulated the Keap1-Nrf2-ARE pathway having led to expression of antioxidant enzyme, inhibiting the oxidative stress, and repairing the damaged proteins [74].

**Figure 3:**
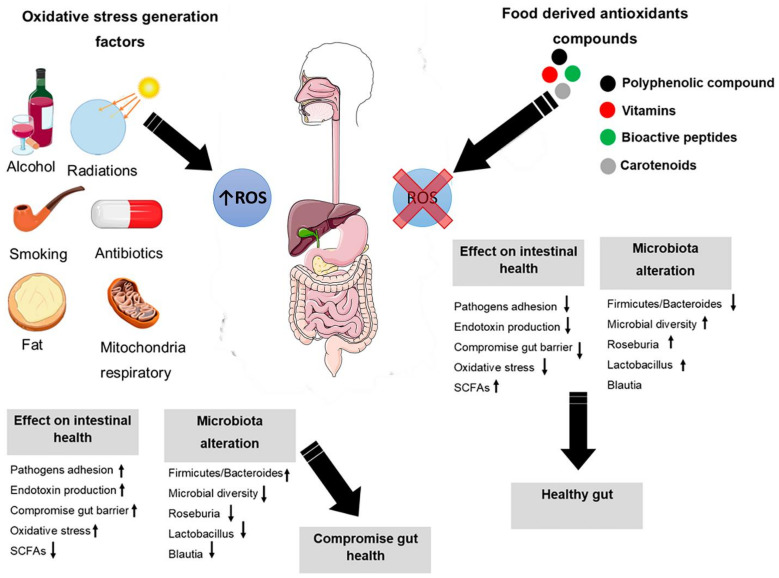
ROS production impact on the homeostasis of host gut. In balance stage, there is a relationship between gut, gut microbiota, and ROS which maintain the function host immune and mucosal defense. In imbalance stage, the over-production of ROS induces oxidative stress which leads to dysbiosis in gut microbiota. The image was created by using the service provide by Servier Mediacl Art under license Creative Common Attribution 3.0 France. ↑: Increase; ↓: Decrease.

The composition of gut microbiota and gut cells are directly correlated with the ROS production in host body. Therefore, ROS might be considered as a double edge sword in the host gut [75]. Thus, there is a complex equilibrium between the production of ROS and the immune responses [76]. Furthermore, under healthy conditions there is a dynamic equilibrium state between ROS formation and elimination from the host body (Figure 3). In addition, the ROS harbor a very significant microbicidal machinery amongst innate cells [77]. The imbalance between the production of ROS and antioxidants will cause oxidative stress which leads to disruption of redox signals and intestinal damage [78].

Furthermore, the consumption of inorganic nitrate exerts a protective effect against oxidative stress via exogenous NO^3−^/NO^2−^/NO pathway and preservation of host gut microbiota homeostasis [79]. Moreover, gut microbiota produces numerous compounds (GSH, butyrate, and folate) beneficial to host intestinal health due to their antioxidant properties and regulation of host intestinal flora. It has been indicated that the host microbiota symbiosis can be regulated via personal nutrition concept built on the results of microbiota analysis [64].

It is very well known that the composition of host gut microbiota can be influenced by a multitude of environmental variables such as diet and infections. A study was carried out to explore the variation in the gut microbiota of the house mouse (*Mus musculus*) on the Isle of May (island off the east coast of Scotland) is associated with environmental and biological factors. This study was particularly designed to investigate the effects of environmental variables, specifically trapping location and surrounding vegetation, as well as the host variables sex, age, body weight and endoparasite infection, on the gut microbiota composition across a fine spatial scale in a freely interbreeding population. The results suggested that the differences in gut microbiota composition were significantly associated with the trapping location of the host, even across this small spatial scale. Furthermore, the sex of the host showed a weak association with microbiota composition. On the other hand, although the sex and location could be identified as playing an important role in the compositional variation of the gut microbiota, 75% of the variation remains unexplained. Finally, it was concluded that fine spatial scales are important when considering gut microbiota composition and investigating differences among individuals [80]. Another study was conducted on the relative importance of geographical location and ethnicity on the gut microbiota composition. The results suggested that both geographical location and ethnicity were the main factors influencing the composition of host gut microbiota. Furthermore, it was suggested that the geographical location exhibited greater influence than ethnicity on both the composition and diversity of the gut microbiota in Chinese population [81]

A study was conducted to compare the composition of the gut microbiome (in terms of gut microbial species richness and abundance) of *Scylla serrata* collected from wild sites and farms, from the east and west coast of India. The effects of the environment on the composition were also tested. The results indicated that the water temperature had a statistically significant effect on gut microbiome composition, with microbial biodiversity decreasing when water temperature increased. This response could have negative effects on both wild and farmed mud crabs under future climate change conditions, although further research into the effects of temperature on gut microbiomes are required. By comparison, salinity, crab mass and carapace width, geographical location as well as whether they were farmed, or wild-caught crabs did not have a significant impact on gut microbiome composition. In addition, the results indicate that farming does not significantly alter the composition of the gut microbiome when compared to wild-caught crabs [82].

It is well known the neurodegeneration by tryptophan metabolites that link the metabolic alterations to Alzheimer’s disease (AD). Furthermore, the tryptophan is a product of Shikimate pathway and the human cells lack Shikimate pathway, which is found in human gut bacteria exclusively. They use Shikimate pathway to produce aromatic amino acids [83]. A recent study was conducted via gene-targeted analysis of human gut microbiota in fecal samples of AD patients. The newly designed oligonucleotide primers were used to target Shikimate pathway-aromatic amino acid in environmental bacteria associated with human activity. The results indicated that a unique gut bacterial sequence was found in most Alzheimer’s disease patients (18 of 20), albeit it was rarely in controls (1 of 13). Furthermore, the cloning and sequencing AD-associated PCR products enables identification of Na(+)-transporting NADH: ubiquinone reductase (NQR) in *Clostridium* sp. The AD-associated PCR products of unrelated Alzheimer’s disease patients possess near identical sequences. The NQR substrate, ubiquinone, is a Shikimate pathway product and human neuroprotectant. In addition, it was revealed a sequence similarity (up to 97%) between AD-associated PCR products and some healthy individuals from different geographical locations. Finally, it was concluded that there were significant differences in the gut microbial genotypes between the AD and control human populations, which is a breakthrough [84]. Hence, the dietary supplements such as vitamins and peptides have excellent potential for nutritional development because of their rich capacity and outstanding properties that regulate the bacterial composition of the gut and the imbalance of redox.

## 7. Dietary Polyphenols on Human Gut Microbiota

This section covers the interaction of dietary polyphenols and their metabolites on the human gut microbial colonization. To date, most of the research on polyphenols is focused on the antioxidant and anti-inflammatory properties but the attention is drawn towards the role of polyphenols on intestinal microbial colonization. Polyphenols’ interaction in several metabolic pathways resulted in the release of metabolites which have shown a positive effect on the gut microbiota by promoting the growth of useful bacteria. Polyphenols are involved in some of the metabolic pathways related to cardiovascular disease and type 2 diabetes prevention pathways [85]. Koudoufio et al. have reported that DNA-based tools helped in the better understanding of the role of polyphenols and their metabolites on the gut microbial ecology [86]. The gut microbes can transform the polyphenols into bioactive metabolites influencing the intestinal ecology [87]. A two-way synergistic effect of polyphenols inhibiting harmful gut bacteria as well as helping bacteria in the enhancing bioavailability of polyphenols were reported by Corrêa et al. [88]. Few pathogenic bacterial species such as *Salmonella* and *Helicobacter* [13], and other harmful bacterial species such as *Clostridium* are inhibited by the polyphenols and in addition, promoted the growth of beneficial *Lactobacillus* species [88]. The authors have also stated that polyphenols maintain gut health by regulating microbial composition. They play a role when directly absorbed in the small intestine or the unabsorbed polyphenols are metabolized by gut microbiota which has beneficial effects in improving host health [89]. In intestinal microbial colonization, the polyphenols promoted the growth of Gram-positive bacteria compared to Gram-negative bacteria [90]. Singh et al., (2019) reported that the prebiotic activity of polyphenols and their metabolites modified the microbial community of the gut [87]. However, the physiological effect depends on the chemical structure, bioavailability, absorption, and metabolism. Polyphenols intestinal absorption is estimated by the enhanced antioxidant defense mechanism [13].

Polyphenols are the secondary plant metabolites abundantly from the food sources such as tea, coffee, wine, cocoa, spices, several fruits and vegetables. Of those sources, fruits and vegetables constitute the highest content of polyphenols. The average intake of polyphenols in many parts of the world is 900 mg/day [91]. Sorrenti et al. reported that the absorption of polyphenols in the small intestine is around 5–10%, whereas the remaining amounts become metabolized by the gut bacteria in the colon [92].

Polyphenols are well known for their antioxidant properties but from the last decade, the interaction of polyphenols and gut microbiota is widely examined. Based on the chemical structure, the polyphenols are divided into phenolic acids, flavonoids and non-flavonoids. Polyphenols mainly exist in the form of aglycones and glycones and a few in the form of condensed tannins. Phenolic acids, which constitute the main part of polyphenols, are divided into benzoic acid and cinnamic acid groups [92]. The group of flavonoids is classified under six sub classes, i.e., flavanols, flavones, isoflavones, flavonols, flavanones and anthocyanidins. The higher molecular weight polyphenols are slowly absorbed when compared to low molecular weight polyphenols [86]. The abovementioned authors have also reported that the dimeric and multimeric polyphenols undergo transformation via hydroxylation, demethylation, decarboxylation, and dehydroxylation by the gut microbiota. Sorrenti et al. reported that the aglycones are easily absorbed in small intestines but for the better absorption of the glycosides, firstly they are hydroxylated by the intestinal microbial enzymes [92]. Similarly, the aromatic ring of the phenolic acid is destroyed by the colonic bacteria releasing the aglycones which can be easily absorbed [93]. Catalkaya et al. also reported that the gut microbe multi-enzymatic reactions lead to the catabolic transformation such as C-C breakdown of the aromatic ring of polyphenolic compounds [94]. The gut microbiota degraded the complex polyphenols to lower molecular weight compounds such as epicatechin and 4-hydroxybenzoic acids [95]. For instance, Marín et al. reported that the flavonoids having linked to sugar moieties must reach the colon, where the colon microbial enzymes hydrolyze them for better absorption. The abovementioned authors have also stated that the polyphenols esterified with sugar moieties and other components require enzyme esterases for the breakdown. Due to humans lack the enzyme esterases, the colon microbial esterases are the main responsible for the breakdown of ester linkages [96]. Similarly, the enzymes such as lactase, phlorizin hydrolase and β-glucosidase released by the gut microbes hydrolyze glycosylated flavonoids, being helpful in absorption. Some of the bacterial species in the intestinal colonization related to polyphenol metabolism are *Enterococcus*, *Streptococcus*, *Lactobacillus*, *E. coli* and *Bifidobacterium* [88].

Polyphenols undergo complex metabolic changes when interacted with microbial enzymes in the gut which resulted in the production of metabolic and catabolic products. In turn, the formed metabolites induce modulation and regulate gut colonization. The interaction between the gut microbiota and polyphenols can be investigated from in vitro and in vivo studies. A smaller portion of polyphenols are absorbed in the intestinal portion of GI tract, where intestinal microbial enzymes also play a significant role in the absorption of polyphenols. Cheng et al. reported that in vitro gut models, which ranged from simple to advanced models, can be applied to analyze the different metabolites formed in the human gut and intestinal microbiota [97]. The in vivo analyses showed that resveratrol is preceded to piceid by the gut microbiota enhancing its bioavailability and antioxidant properties [90]. Kemperman et al. carried out studies on the interaction of tea polyphenols and gut microbiota using a simulator of the intestinal ecosystem. The authors observed that the interaction enhanced stimulation of *Klebsiella* and *Enterococci* but showed an antimicrobial effect on *Bifidobacteria*, *Coccoides* and *Anaeroglobus* species [98]. The prebiotic potential of polyphenols decreased the Firmicutes and Bacteroidetes content in the colon while promoted the host’s health [90]. Mayta-Apaza et al. reported that the prebiotic effect of chlorogenic acid modulated the growth of *Bifidobacterium* [95]. A similar finding was reported by Sorrenti et al. [92], who observed that flavanols modulated the growth of *Bifidobacteria* and *Lactobacillus* and decreased the *Clostridium histolyticum*. Moorthy et al. reported that the polyphenols can bind to its bacterial cell membrane of the *Clostridium* species attributing to this capacity its antimicrobial property [99]. Tea catechins have also showed the suppression of *Clostridium histolyticum* and promoted the growth of *Blautia coccoides* and *Bifidobacterium* [89].

In the investigation carried out by Peng et al. on the effect of cocoa polyphenols on the growth of probiotic bacteria, they observed an increase in the growth of beneficial bacteria such as *Lactobacillus* and a decrease in pathogenic bacteria such as *E. coli*, *S. typhimurium* and *L. monocytogenes* [100]. Epigallocatechin gallate (EGCG) also showed inhibitory action against bacterial species such as *Bacillus*, *Clostridium*, *Streptococcus* and *Salmonella* in the gut [101]. In another study, Murota et al. reported that quercetins are metabolized by gut fecal bacteria such as *Clostridium* and *Bacteroids* to dihydroxylphenylacetic acid (DOPAC) which is a metabolite of potent neurotransmitter dopamine [102]. In an in vivo study conducted by Mayta-Apaza et al. about the role of tart polyphenol consumption on gut microbiota, the authors observed a decrease in the bacterial species such as *Bacteroides*, *Parabacteroides* and *Alistipes* [95].

Moorthy et al. reported that the dietary intake of pomegranate juice extract significantly increased the intestinal *Gordonibacter* composition due to the formation of urolithin which is a breakdown metabolite of ellagitannins by the gut microbiota [99]. In another study, Selma et al. reported that urolithin is a dibenzopyranone metabolite which showed in vivo anti-inflammatory activity [103].

On the other hand, Istas et al. analyzed the composition of gut microbiota using 16S rRNA sequencing tool using double-blind placebo study after the intake of aronia berry polyphenols. The aronia berry polyphenols have modulated the gut microbiota by significantly increasing the growth of *Anaerostipes* and *Bacteroides*. *Anaerostipes* are gut Gram-positive bacteria known to protect from colon cancer [104]. *Duda-Chodak* estimated the minimum inhibitor concentration (MIC) of different polyphenols on the human gut microbiota such as *Bacteroides*, *Lactobacillus*, *Enterococcus*, *Bifidobacterium*, or *E. coli* at different concentrations. From the experimental results, the authors observed that quercetin had the highest inhibitory potential against human gut bacteria compared to other flavonoids such as naringenin, hesperidin, rutin and catechin [105]. 

In another study reported by Loo et al., (2020) on the MIC of quercetin (i.e., concentration ranging from 62.5–250 µg/mL) on the gut microbiota, the in vitro study results showed a decrease in *E. coli*, *Staphylococcus* and *Salmonella*. However, rutin at 10 µg/mL concentration had a significant increase in the growth of *Bifidobacterium* spp. [106]. The consumption of red wine, rich in anthocyanins, has led to increase *Lactobacillus* and *Bifidobacterium* improving gut integrity [99]. In another study on the effect of red wine (600 mg/L) consumption on gut microbiota growth, it was observed that there is no significant increase in growth of gut microbiota in the human fecal bacteria batch-culture fermentation [106]. This shows that the concentration of the polyphenolic extract plays an important role in modulating the gut microbiota. In other fecal bacteria batch-culture fermentation studies, the grape seed extract stimulated the growth of *Lactobacillus* and *Enterococcus* spp. and decrease the *Clostridium histolyticum* bacterial population.

From the in vivo study conducted by Zorraquín et al., it was observed that the intake of wine or grape polyphenols decreased the Firmicutes/Bacteroidetes ratio [107]. The consumption of 1 g/kg diet per day of pure chlorogenic acid stimulated the growth of *Lactobacillus* and decreased the *E. coli* bacteria in the gut of piglets [108]. Similarly, the consumption of chlorogenic acid at 60 mg/kg increased the content of Bacteroidetes [109]. In another in vitro study of olive oil polyphenols on the gut bacteria reported by Merra et al., the polyphenols showed an inhibitory effect of *Helicobacter pylori* at very high concentration [110]. Blueberries extract rich in chlorogenic acid and quercetin (112.5–900 mg/mL) inhibited the growth of *L. monocytogenes* and *S. enteritidis* [111]. From the above experimental results and reported data, it was observed that the intake of dietary polyphenols modulated only a few bacterial species such as *Bifidobacter* and *Lactobacillus*, which are considered beneficial to human gut health (Table 2). Many of the polyphenols have shown an inhibitory effect on harmful bacterial species such as *Clostridium* and *Bacteroides*.

## 8. Gut Microbiota and Antioxidant Vitamins 

The nutrients having antioxidant potential can stop the production of free radicals along with stabilize and scavenging of prevailing free radicals in the host body [124]. The free radical can cause oxidative chain reaction which ultimately leads to cell death [125]. The bioactive substance with antioxidant potential interrupt these chain reactions via modulating the oxidative stress associated with peritoneal diseases [126]. Recently, various studies exploring the role of antioxidant substance in inflammatory disease [127,128]. Some of the most important antioxidant substances found in animals are vitamins. After suffering from stress, an appropriate intake of vitamins significantly reduced the level of oxidative stress along with increasing the antioxidant capacity [79].

Almost 49% vitamins synthesis associated pathways are found in Firmicutes, 19% in Proteobacteria, 14% in Bacteroidetes, and 13% in Actinobacteria phyla [129]. The genome of 256 common bacteria found in gut have been assessed for the presence of vitamins (B1, B2, B3, B5, B6, B7, B9, B12) synthesis pathways and results indicates that these vitamins are synthesized by 40–65% of host gut bacteria [130]. It was also thought that the gut microbiota had the ability to exchange the vitamins with each other, a characteristic which looks vital for those bacterial species having not able to produce those vitamins. The prototrophic bacteria that inhabit the large intestine can produce vitamins de novo [131]. The auxotrophs relaying on prototrophs bacteria for vitamins supply and dietary compounds absorbed in the small intestine of host. Furthermore, the cross feeding between prototrophs and auxotrophs bacteria plays a vital role in maintaining the host gut microbiota homeostasis [132].

The most synthesized vitamins by gut microbiota are riboflavin and niacin having 166 bacteria phyla riboflavin producing and 162 of niacin producing bacterial phyla. In addition, it was estimated how much approximately daily reference intake for the synthesis of these vitamins could be provided from host gut microbiota. The results showed that approximately daily reference intake would be provide in synthesis of pyridoxine (86%), pantothenic acid (0.78%), folate (37%), thiamine (2.3%), cobalamin (31%), riboflavin (2.8%), niacin (27%), and biotin (4.5%) [130]. Furthermore, in adults, the supplementation of *Bifidobacterium adolescentis* DSM 18350, *Bifidobacterium adolescentis* DSM 18352, and *Bifidobacterium pseudocatenulatum* DSM 18353 significantly enhance the concentrations of folic acid in their feces [133,134]. On the other hand, it was shown that the antibiotic treatment exerts negative impression on vitamins synthesis by gut microbiota [135]. It is worth noting that coprophagy provides vitamins that may be absent in an animal diet and preventing coprophagy in a vitamin-deficient diet is a way to adversely affect animal growth, health and survival [136].

The growing evidence suggested that the vitamins derived from gut microbiota play an important role in dysregulation of vitamins-microbiota frontiers, being associated to various diseases [137]. For example, germ free mice having vitamin deficiency prolonged prothrombin time with hemorrhages and 100% mortality, whereas on the other hand all abovementioned abnormalities were not found in reared animals [138].

The vitamins play an important role to control the immune response of microbiota, influencing the gut microbiota composition or leading to microbiota dysbiosis [139]. It was suggested that the treatment of mice with vitamins enhanced the abundance of Citrobacter rodentium via impairment of Th17 response [140]. Furthermore, the parental supplemented with vitamins showed statistically negative trends in abundance of Bifidobacteria, while on the other hand it showed positive trends in abundance of Bifidobacteria fragilis [141]. The growth of Th17 cells is influenced by signals mediated by IL-6 and TGF-β, IL-21, and IL-23 and by stimulation of the lineage-specifying transcription factor, retinoic acid- related orphan nuclear receptor. Th17, which secrete cytokines such as IL-22, IL-17 A, and IL-17 F, are essential in the gut immune homeostasis and inflammation [142]. Vitamins could inhibit and partially reverse experimental autoimmune uveitis by reducing IL-17 production. Additionally, it was proved that vitamin might suppress IL-17 A induction in mouse models of 2,4,6-trinitrobenzene sulfuric acid (TNBS) colitis and early rheumatoid arthritis [143,144]. The administration of vitamins increased the abundance of genera (Akkermansia, Lactobacillus, Parvibacter, Staphylococcus, Corynebacterium) positively correlated with SCFAs. Among these, the abundance of genera Akkermansia, Lactobacillus, and Parvibacter is also positively correlated with the levels of mucins. In addition, the decrease in the abundance of pathogenic Escherichia/Shigella and the increase in the abundance of Akkermansia, Lactobacillus, and Parvibacter might be the key mechanisms underlying the therapeutic effects of vitamin A supplementation on colitis. Such change in gut microbiota might also result in the increase in gut barrier via enhancing the expression of mucins, ZO-1, and anti-inflammatory factors. In addition, the increase in the levels of intestinal SCFAs might also induce the expression of anti-inflammatory cytokine IL-10, promote the growth of therapeutic genera, and inhibit the growth of pathogenic genera in gut microbiota [145].

Recently it was concluded that the higher levels of dietary vitamins for 85-wk-old laying hens improved the production performance and egg quality, which is highly correlated with intestinal microbial. Greater dietary vitamins content contributed to enhanced antioxidant ability, immunity, as well as improved composition of gut microbiota, referring to increased beneficial bacteria abundance, in particular Lactobacillus in the ileum along with Megamonas and Phascolarctobacterium in the cecum. Higher dietary vitamins supplementation was a feasible nutritional strategy to improve the health and production performance of aged laying hens [146]. Hence, it was concluded that the vitamins play an important role in host physiology, therefore our focus will be on antioxidant vitamins.

### 8.1. Vitamin C

Vitamin C, also known as ascorbic acid, is a water-soluble vitamin acting both as an antioxidant and co-factor to modulate several biosynthetic pathways in host immune system [147]. It can be considered as a powerful antioxidant vitamin with excellent scavenging abilities in host body [148], being found in various food within the range of 10–100 mg/100 g. Vitamin C occurs in higher amount compared to other vitamins due to its simple biosynthetic pathway and because of its precursors are sugars, being abundant in most of the diets [149]. Moreover, it is the only vitamin for few vertebrates which lost the capacity to synthesize it. Furthermore, it is a rare compound having hydroxyl group which is completely dissociated at neutral pH due to its acidic nature.

The antioxidant ability of vitamin C is derived from its capability to denote electrons and protect the host from oxidative damage. Vitamin C plays a major role in maintainer skin epithelial barrier along with the function and regulation of host immune system [150]. It is intracellularly accumulated in leukocytes and neutrophils depending upon the availability on the plasma [151]. Furthermore, in neutrophils vitamin C impact the chemotaxis process and phagocytosis process of microbes. In addition, it has antioxidant and scavenging properties that protect neutrophils and phagocytes from damage caused by oxidation explosions, promote programming apoptosis, and activate cascades that inhibit necrosis [152,153]. Vitamin C also improves the antagonistic effect of heat stress and improve lipid peroxidation in broiler [154].

The vitamin C deficiency relates to respiratory tract infection and pneumonia. Hence, during infection, the oxidative stress is elevated. Therefore, the antioxidant potential of vitamin C might be most prominent [155]. Increased demand for antioxidant compounds by white blood cell can generally explain the decline in vitamin C levels observed during infection, especially in patients with lung infections [156] and severe illness [157]. Furthermore, supplementation of vitamin C with thiamin significantly reduced liver cell damage caused by the oxidative stress in mice [158]. Vitamin C reduced ROS generation along with enhancing the activities of SOD and glutathione peroxidase in rat liver mitochondria [159].

### 8.2. Fat-Soluble Vitamins

Vitamin A is a fat-soluble vitamin which can be mainly obtained from animal (retinol) and plant sources (provitamin A carotenoids) [160]. A recent study suggested that the supplementation of vitamin A to DSS mice model exhibited much more abundant gut microbial diversity and flora composition. Furthermore, the metabolomics analysis revealed that the increased production of SCFAs in vitamin A treated DSS mice [161]. In infants, it was suggested that the *Bifidobacterium* and *Bacteroides* contents were positive correlated with the level of vitamin A. Furthermore, the vitamin A deficiency can caused impaired colonic development in GF mice, down-regulated colonic tight junction–related proteins occludin and claudin-1, and reduced immunoglobulin A secretion [162].

In addition, it was suggested that vitamin A deficiency induces colonic inflammation. Colitis is amplified by deficiency of vitamin A and ameliorated by supplementation of the vitamin A [163]. It was suggested that vitamin-A deficiency modified the maturation and differentiation processes of the small intestinal mucosa at the transcriptional and post-transcriptional levels respectively. This, in turn, may be one explanation for the alteration or elimination of nutrient digestion and absorption during vitamin A deficiency [164].

A study investigated the role of β-carotene from Spirulina platensis on growth, serum biochemical, digestive enzymes, antioxidant defense, immune responses, and immune gene expression in Nile tilapia (*Oreochromis niloticus*). The results suggested that the transcripts of interferon gamma and interleukin 1β genes were significantly up-regulated in the liver of fish fed diet supplemented with β-carotene, but expression of HSP70 gene down-regulated in fish fed β-carotene containing diet compared control [165]. Similarly, another study suggested that the β-carotene may provide relieve to tert-butyl hydroperoxide-induced oxidative stress by decreasing reactive oxygen species production and enhancing antioxidant enzyme activities in Caco-2 cells [166].

In another study, it was suggested that the apricot or β-carotene treatment may protect the impairment of oxidative stress and ameliorate MTX-induced intestine damage at biochemical and histological levels [167]. Furthermore, it was suggested that the α-tocopherol, ascorbic acid, and β-carotene, when given concurrently, have primarily antioxidant effects on lipids under stress but do not significantly affect the regulation of p53 gene expression [168].

It was suggested that gut microbiota plays a vital role in regulating the host physiology. Furthermore, the provitamin A (β-carotene) play a positive impact on the reproduction of sows; but still there are very limited study on the influence of provitamin A (β-carotene) on composition of gut microbiota in pregnant sows. A recent study investigated the influence of β-carotene on the production performance of sows from the aspect of gut microbiota. The results suggested that the β-carotene lower the composition of Firmicutes such as *Lachnospiraceae* AC2044 group, *Lachnospiraceae* NK4B4 group, as well as *Ruminococcaceae* UCG-008, but on the other hand significantly enriched the Proteobacteria and Actinobacteria having related to NO synthesis. It was concluded that the supplementation of dietary β-carotene might rise antioxidant enzyme activity and NO which is an important to promote the neonatal blood circulation, through regulating gut microbiota in sows [169].

The weaning is a vital stage in mammals’ life, having associated with intestinal inflammation, causing gut disorders leading to death. It was suggested that the β-carotene exerted the antioxidant as well as anti-inflammatory effect via modulating the composition of gut microbiota. A recent study suggested that the β-carotene improved the growth performance, intestinal morphology and relieved inflammation. Furthermore, it significantly decreased the Bacteroidetes, *Prevotellaceae*, and *Blautia*, and increased the Firmicutes and Parabacteroides in treated groups as compared to control group of animals. Therefore, it was concluded that the β-carotene might have potential to treat the intestinal inflammation via regulating the composition of gut microbiota [170].

It was very well known that the gut microbiota dysbiosis is associated with inflammatory diseases and metabolic syndrome. Recently, it was suggested that the dietary carotenoids such as β-carotene play a vital role in maturation of gut immune system alongside the production of immunoglobulin A, and the consequent promotion of the gut health [171]. It is very well known that the β-carotene have a great potential to display antioxidant and anti-inflammatory activities which in turn provide protection against the development of cancer. On the other hand, still there is unclear about the potential of β-carotene to regulate the composition of gut microbiota and development of ulcerative colitis (UC), which is a kind of inflammatory bowel disease. A recent study investigated the influence of β-carotene on the composition of gut microbiota in a rat model of UC induced by dextran sulfate sodium (DSS). The results suggested that increased the abundance of Faecalibacterium, which is a potential target in the alleviation of DSS-induced UC [172].

Vitamin E is a fat soluble and primary antioxidant of host body. It showed an excellent ability to reduce the oxidative stress and slow down the pathogenesis of several diseases [173]. It consists of eight natural forms having four tocopherols (α, β, γ, δ) and four tocotrienols (α, β, γ, δ). However, the α-tocopherol is known to be the most abundance and efficient one in hindering the oxidation of lipid [174]. Vitamin E significantly reduced the expression of TGF-β and lipid peroxidation via stimulation of hepatic stellate cells [175]. It is also known as powerful antioxidant of lipoprotein and cell membrane owing to its fat-soluble stuff [176].

Vitamin E exerts excellent antioxidant activity via scavenging lipid peroxyl radicals through providing a hydrogen ion from its chromanol ring. In addition, vitamin E acts as a strong scavenger of superoxide, peroxyl, and hydroxyl radicals to provide protection against lipid and lipoprotein peroxidation [177,178]. Furthermore, it also shows excellent ability to scavenging the reactive nitrogen species [179]. Thus, the intake of higher amount of vitamin E will be very helpful to reduce the risk of oxidative stress in host body. For instance, vitamin E interaction with cellular components might be very helpful to foster the anti-oxidative environment in the host body [180]. The superoxide dismutase is an enzyme, which break down the superoxide radical into hydrogen peroxide and oxygen. The daily intake of vitamin E will be very helpful to stimulate the level of superoxide dismutase [180]. Furthermore, the vitamin E also stimulate the action of various anti-oxidative enzymes such as catalase and peroxidase [181]. The vitamin E also reduced alcohol-induced oxidative stress in small intestine of rats along with the improvement of total anti-oxidative capacity and reduction of oxidative stress. Moreover, it was also helpful to reduce the inflammatory markers such as IL-6 and TNF-α [182]. In addition, vitamin E has shown an excellent potential to attenuate the colitis, but the mechanisms are not fully understood. In this line, a recent study investigated the impact of α-tocopherol and γ-tocopherol-rich tocopherols on gut inflammation, barrier integrity and microbiota in dextran sulfate sodium (DSS)-induced colitis in mice. The results suggested that the specific forms of vitamin E could provide protection effect on intestinal barrier function via altering the composition of gut microbiota in colitis-induced mice [183].

## 9. Bioactive Peptides Activity on Gut Microbiota

The imbalance between the ROS and antioxidant compounds production can cause the gut microbiota dysbiosis and cell injury [184]. Bioactive peptides can reverse the gut microbiota dysbiosis via regulating the ROS production level in host body [185]. Recent studies suggested that some bioactive peptides (NVPVYEGY, SLEAQAEKY, GTEDELDKY, DSGVT, IEAEGE, and DAQEKLE) exert excellent antioxidant activities against oxidative stress [186,187]. Bioactive peptides showed higher antioxidant activity and can be easily absorbed in the intestine as compared to native protein [188]. Peptides exert antioxidant activity by scavenging the ROS and to promote the antioxidant system of gut cells [189].

Moreover, bioactive peptides modulate the composition of gut microbiota, which in turn might affect host gut and immune system. For instance, the quantity as well as source of dietary protein may regulate the metabolites produced by rectal mucosa gene expression and microbiota [190]. Furthermore, the differences in gut metabolites within individuals was linked with level of amino acids amongst individual [191]. The consumption of hydrolyzed proteins significantly reduced gut microbiota dysbiosis by shifting the microbial community via decreasing the abundance of *Clostridium perfringens* and *Escherichia coli* [109]. The consumption of whey protein significantly increased the abundance of *Bacteroidetes*, *Lactobacillus*, and *Bifidobacterium* in rats [192]. The Parmigiano-Reggiano cheese derived peptides enhance the abundance of *Lactobacillus* and *Bifidobacterium* [193]. The above discussion revealed that the peptides efficiently regulate the composition of gut microbiota which may leads to affect the gut and host mucosal immune system. Hence, preparation and identification of functional peptides from food and their impact on the microbiome can lead to a better understanding of the development of functional foods.

## 10. Conclusions

Presently, it has been proven that there is a positive impact of various food derived compounds on the composition of host gut microbial communities; but still a considerable effort is necessary for comprehensive assessment of the impact of food derived antioxidant compounds on the composition of gut microbial communities and their impact on host health. The homeostasis between gut microbial communities and generation of ROS might be altered by food derived compounds. On the other hand, the antioxidant compounds from several food sources improve host health by regulating the composition and configuration of gut microbiota. Hence, these antioxidant compounds can modulate gut microbiota and might be used as novel strategies to control and reduce host intestinal oxidative stress. However, more research is needed, focusing on the bioavailability of antioxidant compounds and their effect on various diseases.

## Figures and Tables

**Table 1:** Common risk factors of intestinal oxidative stress.

Risk Factors	Oxidative Stress Classification	Experimental Model	Intestinal Impact	Mechanism of Action	References
**Herbicides**	Hydrogen peroxide	Rats	Intestinal flora and inflammatory response	Intestinal inflammatory response, α-diversity, pathogenic bacteria, and ↑ ROS	[52]
**Cigarette**	ROO• and carbon centered radicals	Rats	Intestinal dysregulation and ↑ Oxidative stress	α-subunit of the flavocytochrome b558, Claudin-2, Claudin-1, SOD, and bcl-2,	[53]
**Intestinal I/R**	Superoxide	Rats	Alteration in composition of cecal and neutrophil infiltration	Ileum and colon ↑ MDA and ileum MPO	[54]
**γ-Irradiation**	Hydroxyl radicals	Cellular model	Apical junctional complex Disruption and dysfunction of mucosal barrier	Thiol’s protein reduction, ZO-1, Nrf2 and various enzymes of antioxidant	[55]
**Pathogens**	Superoxide	*Salmonella typhimurium*	Dysregulation of microbiota and oxidative stress	Factor relating to inflammation, NF-κB p65 expression, and MPO activity	[56]
**Severe sleep loss**	None	Enterocytes	Lifespan shortening	ROS accumulation and oxidative stress	[57]

ROS: reactive oxygen species; SOD: super oxide dismutase; I/R: ischemia−reperfusion; MDA: malondialdehyde; MPO: myeloperoxidase. ↑: Enhance the activity.

**Table 2:** Effect of dietary polyphenol on the growth of different bacterial species.

Polyphenolic Compound/Extract	Content	Modulation of Bacterial Species	References
**Naringenin and** **hesperetin**	≥250 µg/mL	*Bacteroides*, *Lactobacillus*, *Enterococcus*, *Bifidobacterium*, *E. coli*	[105]
Daidzein and genistein	1000 µg/mL	*E. coli*, *S. typhimurium*, *L. rhamnosus*	[112]
Resveratrol	200 mg/kg/day	*Lactobacillus*, *Bifidobacterium*	[113]
Red wine	272 mL/day	*Bifidobacterium* *C. histolyticum*	[114]
Grape pomace extract	700 mg/day	*Lactobacillus*, *Bacteroides*	[115]
Pomegranate extract	250 mg/kg BW/day	*Lactobacilli*, *Bifidobacteria* *E. coli*	[116]
Apple peel	300 mg/kg/day	*H. pylori*	[117]
Berry extracts	1 mg/mL	*H. pylori*, *B. cereus*	[118]
Catechin	125 µg/mL	*S. aureus*, *S. typhimurium*	[112]
Chlorogenic acid	100 µg/mL	*Bifidobacteria*	[119]
Oil tea	4 g/kg BW/day	*Lachnospiraceae*, *Erysipelotrichaceae* *Lactobacillus*	[120]
Olive oil	1.3 µg/mL	*H. pylori*	[121]
Blackcurrant extract powder	13.4 mg/kg BW/day	*Lactobacilli*, *Bifidobacteria* *Bacteroides*	[122]
Black raspberry powder	5% *w*/*w*	*Anaerostipes* *Acetivibrio*	[123]

BW: body weight.
